# Leakage Source Localization Method for Aerospace Composite Structures Based on U-Array Wave Velocity-Compensated Beamforming

**DOI:** 10.3390/s23135870

**Published:** 2023-06-25

**Authors:** Lei Qi, Lixin Xu, Lichen Sun, Xiaobo Rui, Yuhao Cui, Xin He, Yu Zhang

**Affiliations:** 1 Beijing Institute of Spacecraft Environment Engineering, Beijing 100094, China; 2 State Key Laboratory of Precision Measurement Technology and Instrument, Tianjin University, Tianjin 300072, China

**Keywords:** composite structure, beamforming, U sensors array, sub-array, anisotropy

## Abstract

Composite materials have been widely used in spacecraft structures. Due to the harsh environment in space, gas leakage will occur in the structure, so it is necessary to locate the leakage position in time. In this paper, a beamforming localization method based on a U-shaped sensor array is studied. The array can be divided into two subarrays, which can orientate the direction of leakage sources, respectively. To solve the problem of uneven wave velocity caused by the anisotropy of composite materials, this method modifies the relationship between wave velocity and direction and combines it with the dispersion curve to select a filtering frequency band to reduce the influence of dispersion. The experiment simulates vacuum leakage by pumping holes with a diameter of 3 mm with a vacuum pump. The results show that the U-shaped array beamforming algorithm proposed in this paper can obtain a positioning error of 2.21 cm, which provides a new idea for the structural health detection of spacecraft.

## 1. Introduction

With the continuous development of the space industry, an increasing number of large spacecraft are being put into use. Due to their excellent mechanical properties, composite materials are widely used in new spacecraft structures, for example, space shuttles, rocket fairings, and satellite fuel cylinders. However, during the process of launch, flight, and re-entry, spacecraft face severe space environments, such as vibration and impact, high- and low-temperature alternation, and debris impact. The composite structure will generate local stress and defect expansion and may produce tiny leakage holes, leading to gas leakage and threatening the safety of the spacecraft [1]. Therefore, it is important to locate the leakage source of the composite structure in time to ensure the safety of the spacecraft.

When a leak occurs, an acoustic emission signal will be generated at the leak location and propagate over the bulkhead structure in the form of Lamb waves. However, unlike metal structures, composite structures exhibit anisotropy in their physical properties, which leads to more complex propagation laws for acoustic emission signals. Compared with the traditional optical fiber method [2] and helium mass spectrometry [3], the acoustic emission method is a lightweight and efficient acoustic emission source localization method [4], and many scholars have carried out related research on the localization of composite structure acoustic source signals.

Zhang et al. [5] proposed an improved trigonometry method based on the idea of arrival time and signal intensity synthesis and combined it with the neural network training method to realize the localization of impact signals in the composite structure. Yuan et al. [6] realized the impact signal localization of composite structures through an improved multi-signal classification algorithm. Ren et al. [7] applied the two-dimensional multiple signal classification method to the near-field impact signal detection of composite materials and verified it experimentally on real aircraft composite fuel tanks. Kundu et al. [8,9,10] proposed small sensor array methods based on L-type, square, and Z-type, respectively, to realize the localization of burst acoustic sources in composite structures with unknown material properties. In addition, they put forward a method based on signal energy that establishes the nonlinear equations of sensor and signal source parameters and realizes positioning through the least squares solution [11]. He et al. [12] designed a method based on the Hilbert yellow curve to achieve rapid localization of burst acoustic sources in metal and composite structures. Al-Jumaili proposed a dealt-T mapping method [13], and Xu et al. proposed a frequency domain feature ratio vector matrix index method, which realizes positioning by constructing energy ratio vectors in different frequency bands of the signal and matching the index matching with the frequency domain feature matrix constructed in advance [14]. However, the disadvantage of these methods is that they have a large workload and are difficult to apply to large structures. Ebrahimkhanlou et al. [15] proposed a method based on deep learning that used a sensor to locate and characterize artificial impact sources in complex aircraft fuselage segments. Zhang et al. [16] used the cross-correlation method to locate the liquid leakage source in the fuel storage tank. However, this method is also applied to burst leakage sources. Yuan et al. [17] proposed a localization method based on the MUSIC algorithm for aircraft structural damage detection. It used the Gabor wavelet transform to extract the narrow frequency band of the acoustic source and combined it with the MUSIC algorithm to scan the detection area to detect the distance and direction of the leak source. Wang et al. [18] proposed a method based on an ultrasonic sensor array, which realized the location of the gas leakage source by detecting leakage signals in the air and combining them with the TDOA method.

The beamforming method is a classical acoustic source localization method, different from acoustic cameras, for acoustic emission signals. This method usually uses a one- or two-dimensional array to collect acoustic emission signals and delay the superposition of the signals for each channel to achieve orientation or positioning [19]. Li et al. used time-frequency analysis and narrowband filtering to extract the effective information of the leakage signal and realize the localization of the continuous leakage source of CO_2_ gas through the linear sensor array [20]. The team conducted an in-depth study of beamforming geometric arrays, and the results showed that L-shaped geometric arrays have the best performance in acoustic emission positioning in terms of both sensor number and positioning accuracy [21]. Hayato Nakatani et al. [22] proposed an idea to improve the beamforming algorithm by compensating for the direction and wave speed and locating the burst acoustic source by linear array, but the application of this method for leakage signals is unknown. Zhang et al. compensated for the frequency dispersion problem in the plate structure by calculating the propagation phase velocity of the leakage signal and the spatiotemporal matrix of the leakage signal and realizing the localization of the continuous gas leakage source in the plate structure [23]. Based on the L-shaped array, Qi Lei et al. proposed a frequency-weighted matrix beamforming algorithm, which reduced the influence of the frequency dispersion phenomenon in the plate structure and realized the localization of leakage signals in metal-reinforced plates [24]. However, the above method is mainly aimed at the source localization of metal structures, and the number of sensors is also required. The applicability of composite structures is difficult to guarantee. In addition, the leakage signal is continuous, the arrival time of the signal is not obvious, and the anisotropy of the composite material brings more difficulties to the location of the leakage source. Therefore, it is very important to study the leakage location method for composite structures. To solve the problem of locating leakage sources in composite structures, this paper proposes a beamforming method based on U-shaped arrays. Firstly, the leakage signal in the composite structure is collected by a U-array, and the frequency spectrum of the signal and the dispersion information of the composite structure are filtered by a narrow band and matched by wave velocity. Finally, the signal of each array element is delayed, and energy is superimposed by beamforming to realize the location of the leakage source in a single array.

The rest of the paper is organized as follows: in Section 2, the method flow of the U-shaped array is presented; Section 3 introduces the experimental platform of the laboratory; in the Section 4, the experiments performed to demonstrate the performance of the method are described; and the conclusions are presented in Section 5.

## 2. Method and Process

### 2.1. Method

**Figure 1:**
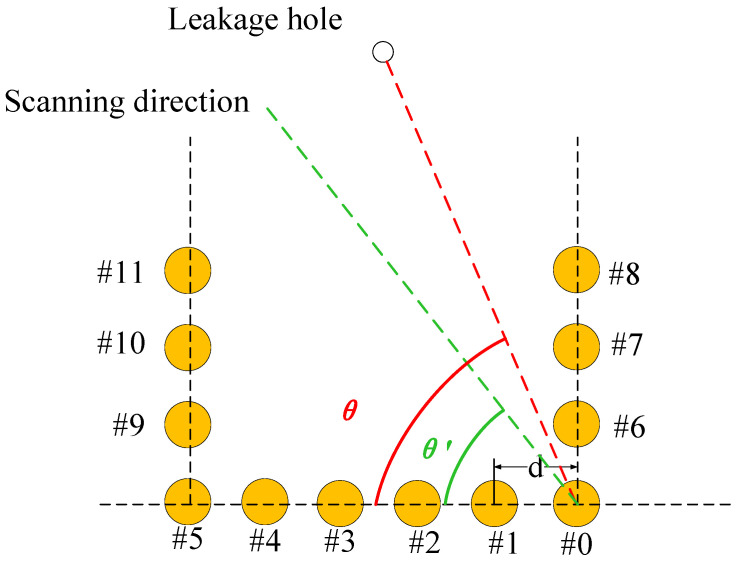
Demonstration of U-array beamforming.

**Figure 2:**
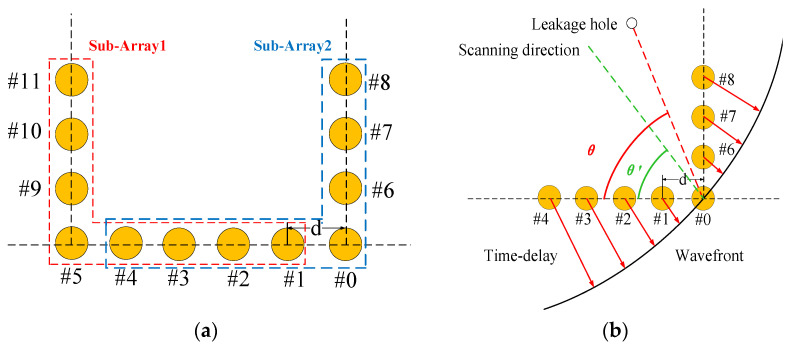
Principle of u-array positioning: (**a**) structure of the subarray; (**b**) principle of subarray beamforming scanning.

In previous studies, the L-shaped array has been confirmed to have a better localization effect than other topological arrays, but the location range is limited, and the leakage source can only be posited in the 0–90° range inside the L-shaped array, and the leakage source location cannot be determined, which limits the engineering application scenarios. The structure of the U-shaped array is shown in Figure 1. Take a U-shaped array of 12 sensors as an example, consisting of one long side and two short sides, although this structure is also suitable for formats with different numbers of sensors. The array can be divided into two L-shaped subarrays, and the subarray consists of 8 sensors, respectively composed of the long side and short side, wherein the position of the #0 sensor and the #5 sensor is used as the reference sensor of the two subarrays, and the line between the leakage source and the #0 sensor position is the angle of the long side as the leakage source direction. Each subarray can individually orient the source of the leak, and the structure of the subarray is shown in Figure 2.

In a thin plate structure, the acoustic emission signal generated by the gas leakage propagates in the plate in the form of Lamb waves. Lamb waves will produce frequency dispersion during propagation. Unlike metal structures, composite structures exhibit anisotropy in their physical properties. When the acoustic emission signal propagates on the composite structure, its frequency dispersion phenomenon will be more complex, and its wave speed and propagation direction are related, so in the process of beamforming algorithm positioning, it is necessary to obtain wave speed information in all directions.

As the Lamb wave signal will generate a dispersion phenomenon when it propagates in the plate structure, it is necessary to calculate the dispersion curve of the Lamb wave, which can be classified into antisymmetric mode (A) and symmetric mode (S).

When the leakage signal propagates through the composite plate, the wavefront can be approximated as a circular arc, where the center of the arc corresponds to the location of the leakage source. The leakage signal *S*_0_(*t*) in the [*f_a_*, *f_b_*] band acquired by the sensor array can be expressed as:

(1) $$S(t)=\int_{f_{a}}^{f_{b}}A(f,t)\exp(j2\pi f)df.$$

where *A*(*f*, *t*) is the amplitude function of the propagation signal, which is related to the frequency and propagation time of the signal, and exp(*j*2*πf*) represents the phase information of the signal. Due to the different positions of each element in the array, a certain time difference will occur when the signal propagates to each element. Assuming that the leakage signal is generated at (*x_s_*, *y_s_*) and the coordinates of sensor #*i* are (*x_i_*, *y_i_)*, the angle between the acoustic source and the individual sensors is

(2) $$\theta_{i}=\arctan\left|\left(\frac{y-y_{i}}{x-x_{i}}\right)\right|,$$

The distance *L* between the acoustic source and the individual sensors is

(3) $$L_{i}{}^{\prime }=\sqrt{{\left(x-x_{i}\right)}^{2}+{\left(y-y_{i}\right)}^{2}},$$

The delay time Δ*t_i_*(*f*, *θ*) between the signal received by each sensor and the signal received by the reference sensor is

(4) $$\Delta t_{i}(f,\theta )=(\frac{L_{0}}{c_{0}(f,\theta_{0})}-\frac{L_{i}}{c_{pi}(f,\theta_{i})}),$$

where *c*_0_(*f*, *θ*_0_) is the phase velocity of F when the frequency is *f* and the propagation direction is *θ*_0_, *c_pi_*(*f*, *θ_i_*) represents the phase velocity of the leakage signal when the frequency is *f* and the propagation direction is *θ_i_*. *L*_0_ is the actual distance between the leakage source and the #0 sensor, and *L_i_* is the actual distance between the leakage source and the #*i* sensor. The leakage signal propagated to the individual elements at this point can be expressed as

(5) $$S_{i}(t)=\mu_{i}S(t-\Delta t_{i}(f,\theta )).$$

where *μ_i_* represents the amplitude attenuation factor.

According to the idea of the beamforming method and the signal delay relationship between each sensor, the signal of each channel is reversed delay processed, so that the signal phase of each channel moves to the same wavefront, and the array signal superimposed by delay can be represented as:

(6) $$g(t,\theta )=\sum_{n=0}^{7}S(t-\Delta t_{i},n)\cdot d(\Delta t_{i}(f,\theta ))+S(t,0),$$

where *d*(*t*) is the inverse delay function of the signal. The signal energy can be obtained by integrating the time-domain sum of squares on the superimposed signal. By scanning the hypothetical angle, we obtain an energy function *B*(*θ*′) concerning the angle:

(7) $$B(\theta ’)=\int g^{4}(t,\theta ’)dt,$$

The output energy is calculated for the superimposed signals in each scanning direction, and the scanning direction of a single subarray traverses the 0–180° direction. After obtaining the spatial energy distribution, the direction of the maximum energy is used as the direction of the leakage acoustic source. When the two subarrays have finished scanning in different directions, the source of the leak can be located. If the coordinates of the #0 sensor and the #5 sensor are (*x*_0_, *y*_0_), (*x*_5_, *y*_5_), respectively, and the orientation results of the subarray are *θ*_1_ and *θ*_2_, respectively, then the leakage source coordinates (*x*, *y*) have the following geometric relationship with the reference element of the U-shaped array:

(8) $$\sqrt{{\left(x-x_{1}\right)}^{2}+{\left(y-y_{1}\right)}^{2}}=\frac{\sin(\theta_{2})}{\sin\left(\theta_{1}+\theta_{2}\right)}L,$$

(9) $$\sqrt{{\left(x-x_{2}\right)}^{2}+{\left(y-y_{2}\right)}^{2}}=\frac{\sin(\theta_{1})}{\sin\left(\theta_{1}+\theta_{2}\right)}L.$$

where *L* is the straight-line distance of the #0 and #5 sensors, and Equations (8) and (9) are solved synchronously to solve the coordinates of the leakage source.

### 2.2. Process

**Figure 3:**
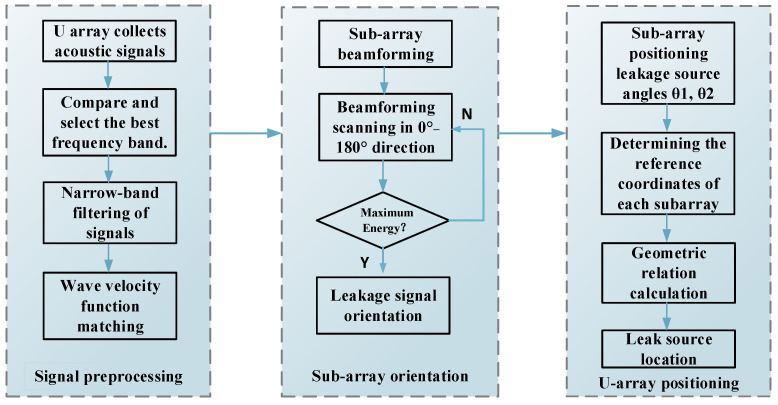
Flow chart of the U-array positioning beamforming method.

Figure 3 introduces the whole process of locating the leakage source using the U-array method, which can be divided into three parts: signal preprocessing, subarray directional processing, and U-array positioning processing.

(1) Signal preprocessing

Step 1: The sensor array is arranged in the composite structure, and after the leakage signal is generated, the leakage signal is collected through the U-shaped sensor array, and the data is saved according to the number.

Step 2: Perform fast Fourier transform processing on the signal, check the leakage spectrum, select a suitable narrow band as the filter band, and filter.

Step 3: According to the center frequency of the filter band, compare the dispersion curves in different directions of the composite structure to match the wave velocity function.

(2) Subarray directional processing

Step 1: Arrange the data of the two subarrays by number.

Step 2: Delay the signal of each channel according to Equations (4)–(7), scan the detection area in the direction of 0–180°, look for the corresponding direction of the energy peak, and complete the subarray orientation.

(3) U-array positioning processing

Step 1: Obtain the orientation results of the subarray.

Step 2: Connect the geometric relationship according to the reference sensor coordinates of the two subarrays, calculate the location of the leakage source, and realize the location of the leakage source.

## 3. Experimental Platform

To verify the algorithm proposed in this paper, an experimental system is designed. As shown in the figure, the experimental system consists of a composite plate, a vacuum pump, a piezoelectric sensor, a signal acquisition system, and a computer. The composite board is a T300/IS1301 epoxy carbon fiber material laminate, its plate thickness is 3 mm, the side length is 1000 mm, the number of laying layers is 15 layers, the laying order is [0_3_/90/0_3_/90/0_3_/90/0_3_], and the composite plate structure is orthotropic as a whole. Other mechanical parameters are shown in Table 1.

**Figure 4:**
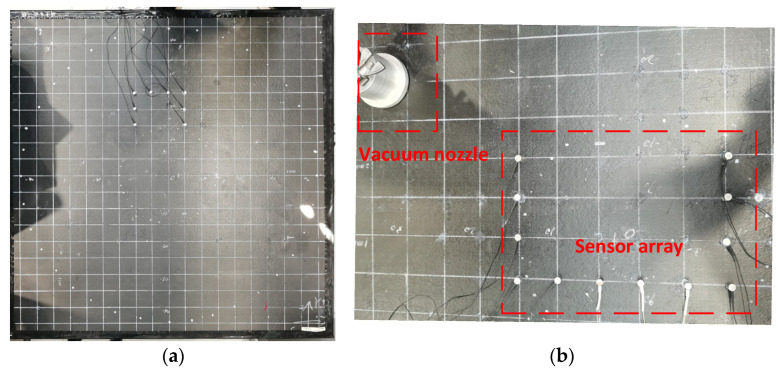
Schematic diagram of the test system. (**a**) Image of the composite structure; (**b**) U-sensor array and leakage source.

The experimental composite board is shown in Figure 4a. To facilitate location and parameter recording, a Cartesian coordinate system is established on the test plate, with the center position as the center of the circle. To reduce the interference of boundary reflection on the positioning method, sound-absorbing cement is pasted on the boundary of the plate. One leak hole with a diameter of 3 mm is machined on the board, and its coordinates are (−35, 20), which can simulate real gas leakage with a vacuum pump. The locations of the leak hole and the sensor array are shown in Figure 4b. The pumping rate of the vacuum pump is 8 L/s, and when working, it is attached to the leakage position through the vacuum suction nozzle, and the vacuum pump is pumped when it is working, simulating the gas leakage of the spacecraft cabin structure in orbit.

**Figure 5:**
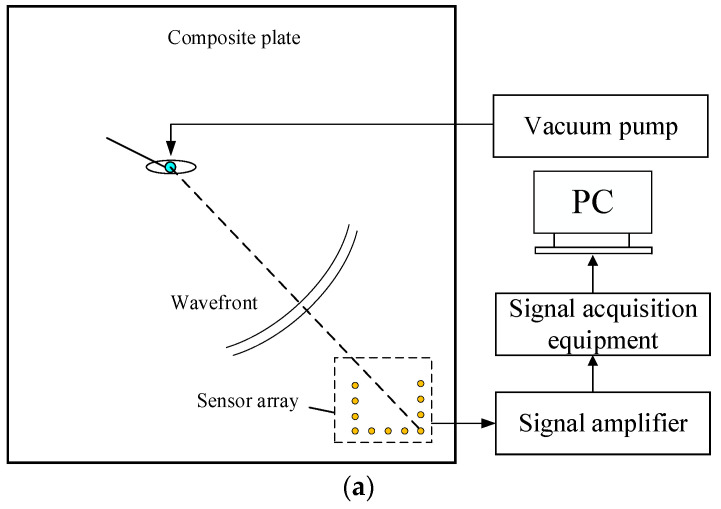

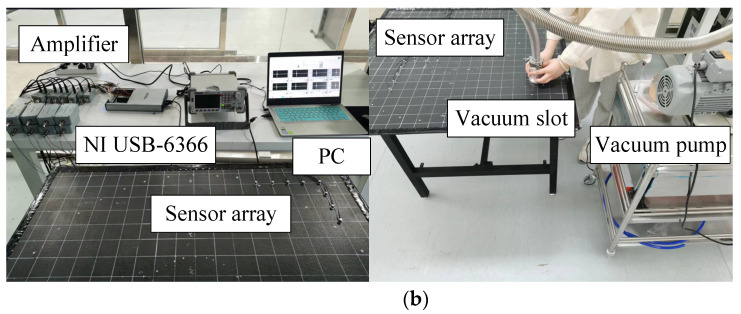
Experimental system. (**a**) Schematic diagram of the experimental system. (**b**) Experimental system diagram.

The sensor array element is a Nano-30 piezoelectric sensor produced by PAC. The U-sensor array consists of 12 identical Nano-30 sensors with a spacing of 5 cm between the sensors. The array structure and the position relationship between the sensor array and the leak are shown in Figure 5a. The sensor signal is amplified by an amplifier, and the amplification gain is 40 dB. The signal acquisition system uses USB-6366 acquisition equipment from NI with a sampling rate of up to 2 MHz. It can realize the synchronous acquisition of 8-channel signals. The relevant acquisition program is written by LabView software to save the sensor signal. The physical diagram of the experimental system is shown in Figure 5b.

**Figure 6:**
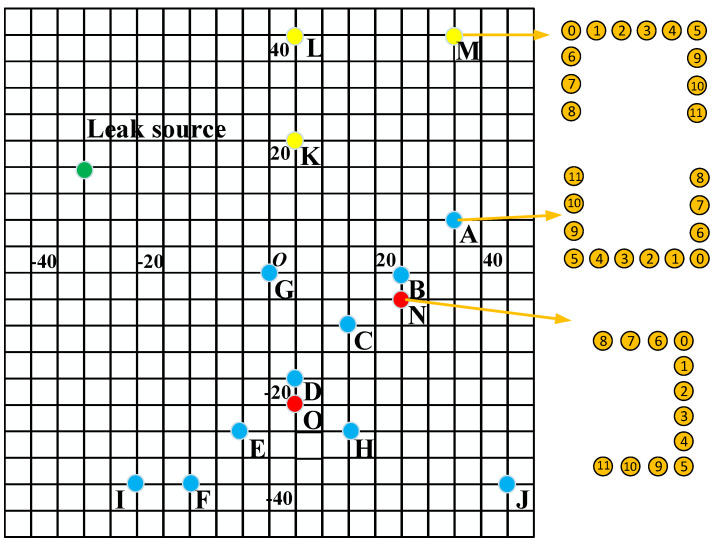
The position distribution of the sensor array and leakage source.

Experimental steps:

In the experiment, the location of the leakage source and the sensor array is shown in Figure 6, where the leakage source is marked with a green circle. The array has been tested in 15 positions, and the array can be divided into three types, which are represented by circles with three colors, in which blue is the reference format, red is the array rotated 90 degrees, and yellow is the array rotated 180 degrees. The position of the #0 sensor of the sensor array is marked with circles with corresponding colors in Figure 6, and Table 2 shows the coordinates and actual directions of each position of the leakage source and the sub-array reference sensor.

## 4. Position Results

**Figure 7:**
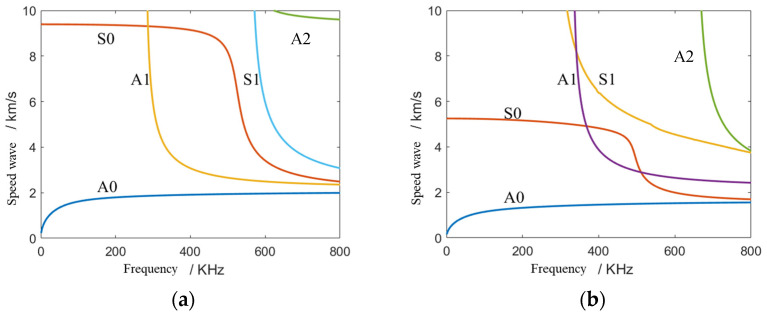
Dispersion curve of phase velocity: (**a**) 0° direction; (**b**) 90° direction.

This section gives an introduction and discussion of the localization results. In isotropic structures, symmetric modal (Mode S) and antisymmetric modal (Mode A) dispersion curves can be solved by the Rayleigh-Lamb equation. However, in composite structures, the Lamb wave mode will not be a simple symmetric mode and an antisymmetric mode. To facilitate understanding and description, the different modes in the composite structure are still described as A mode and S mode in this paper. According to the parameters of the composite structure described in Section 3, the frequency dispersion curve of the composite plate is calculated by MATLAB software, and the frequency dispersion curves of the phase velocity of the composite plate in the 0° direction and 90° direction are shown in Figure 7.

**Figure 8:**
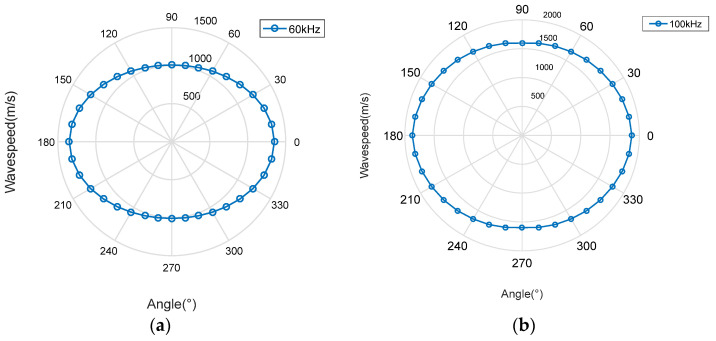
Wave velocity−direction curve: (**a**) 60 kHz; (**b**) 100 kHz.

From Figure 7, only A0 and S0 modes exist in the frequency band below 200 kHz. The energy of the A0 mode is much larger than that of the S0 mode, and the wave velocity of the S0 mode changes greatly, and its stability is poor. Therefore, A0 mode will be selected for signal processing. Taking the A0 mode at 60 kHz and 100 kHz frequencies as examples, the dispersion curve in all directions is calculated and obtained, the phase velocity–direction curve of the A0 mode at 60 kHz and 100 kHz frequencies is fitted and displayed in polar coordinates, and the fitted wave velocity function curve is shown in Figure 8.

**Figure 9:**
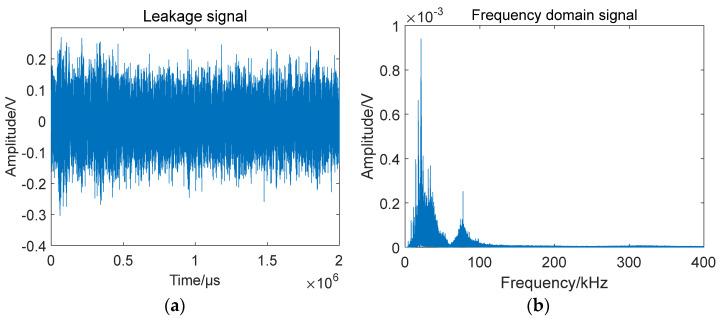
Gas leakage in vacuum: (**a**) signal in the time domain; (**b**) signal in the frequency domain.

From Figure 8, the distribution of Lamb waves in the direction of the phase velocity is elliptical, which indicates that the Lamb wave propagation in the above anisotropic material structure is related to the laying mode of the structure. To select a suitable narrow frequency band as the filter frequency band of the leakage emission signal, it is necessary to view the frequency domain information of the leakage signal. Taking the #0 sensor data of experiment number 1 as an example, the time domain and frequency domain signals of the leakage signal are shown In Figure 9.

From the information in Figure 9, the amplitude distribution of the leakage signal in the time domain is relatively uniform, and there is no obvious arrival time. In the frequency domain, the energy of the signal is mainly concentrated below 40 kHz, and there is a lower energy region in the 40–120 kHz band. Since the acoustic emission signal generated by gas leakage is a broadband signal, to ensure the stability of the wave speed, narrowband filtering of the signal is usually required. According to the dispersion curve, the phase speed of the signal below 40 kHz changes rapidly, and there are many low-frequency noise signals, so the 40–120 kHz frequency band is selected as the selected frequency band for narrowband filtering when performing signal filtering processing. When filtering, the bandwidth of the narrow band is 20 kHz, and the wave velocity function curve is matched according to the wave velocity in the dispersion curve corresponding to the center frequency of the frequency band. The phase velocities corresponding to the 0° and 90° directions for each frequency are shown in Table 3.

**Figure 10:**
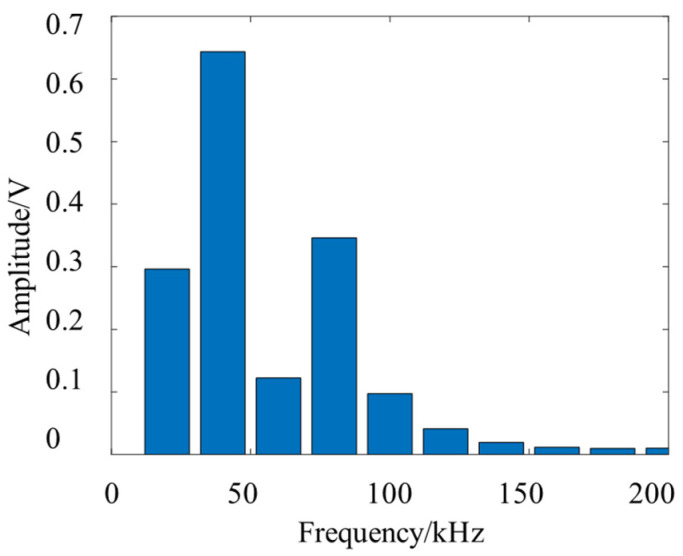
The frequency band energy value of the leakage signal.

Comparing the energy of each band of the leakage signal, the signal band energy value is shown in Figure 10.

According to the dispersion curve, the phase velocity of the Lamb wave signal in the frequency below 50 kHz changes greatly, so it is difficult to apply it to the location of the leakage source, and the energy amplitude of the 70–90 kHz band is the highest in the narrow frequency band above 50 kHz, so this frequency band is selected as the narrow band of signal filtering. Combined with the dispersion curve, the wave velocity-direction curve within this frequency band can be plotted. After determining the filter frequency band and wave velocity function curve, the delay matrix can be calculated by Equations (4)–(6), and beamforming scanning can be performed in all directions to find the corresponding direction of the energy peak and complete the localization.

**Figure 11:**
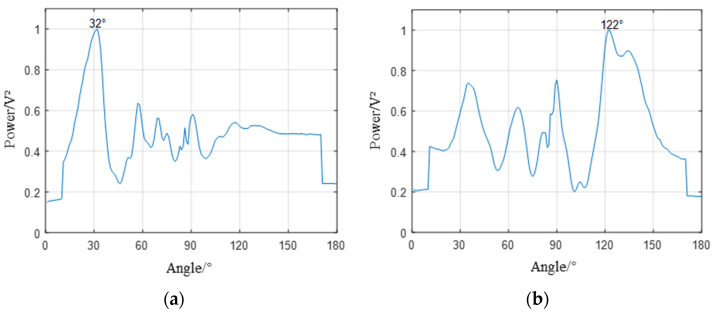
Subarray orientation diagram. (**a**) The orientation result of subarray1 is 32°, and the actual direction is 31°. (**b**) The orientation result of subarray2 is 122° (the actual direction is 126°).

Figure 11 shows the localization results of the two sub-arrays of the U-array to the acoustic source at the C position. According to Table 2, the directional angles of the two sub-array reference sensors and the leakage source are 125° and 31°, respectively, while the directional results of the subarray are 122° and 32°, and the directional error is 1° and 3°, and the directional error is small. It can be seen from Figure 10 that although there are pseudo-peaks in the directional results of beamforming, which proves that the reflection of the signal has a certain impact on the positioning results, the unimodal nature of the directional results corresponding to the peaks is good, which proves that the robustness of the method is good.

**Figure 12:**
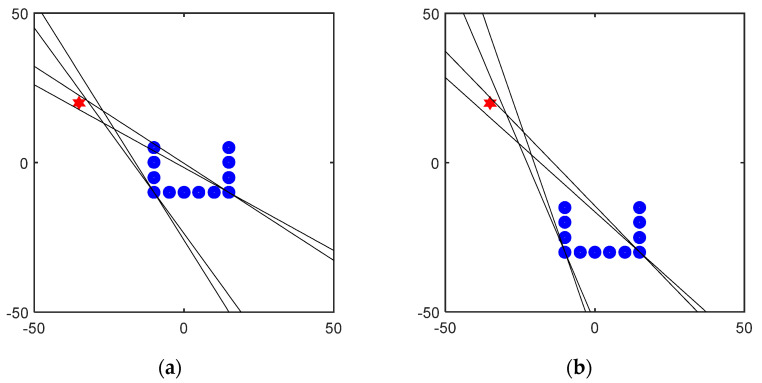

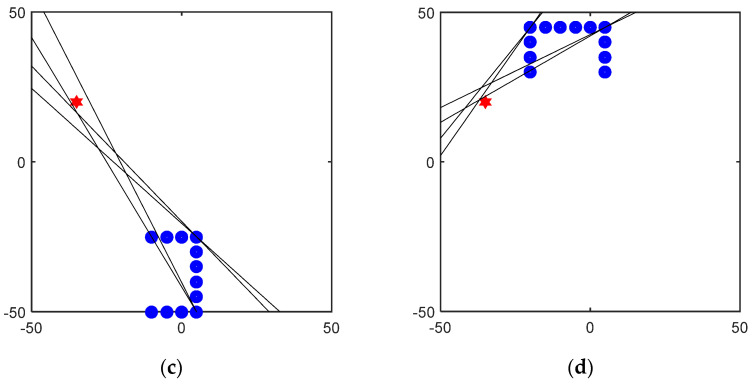
U-array positioning result diagram: (**a**) The positioning results of G point and the subarray orientation results are 31° and 122°. (**b**) The positioning results of the H point and the subarray orientation results are 44° and 111°. (**c**) The positioning results of the O point and the subarray orientation results are 136° and 29°. (**d**) The positioning results of the L point and the subarray orientation results are 28° and 53°. The leak source is marked with a red five-pointed star.

After the leakage source is oriented through two subarrays of the U-array, the leakage source can be located and processed. Taking the measurement results of G, H, O, and L as examples, the positioning results of three types of arrays are demonstrated, respectively. The positioning results are shown in Figure 12. The leak source is marked with a red five-pointed star. After the leakage source is oriented through two subarrays of the U-array, the leakage source can be located and processed. Taking the measurement results of G, H, O, and L points as examples, the positioning results of three types of arrays are demonstrated, respectively. The positioning results are shown in Figure 12.

In the location results, to locate the leakage source, a location area is usually drawn according to the ±2° of the location angle, and the shortest distance between the location area and the leakage source location is used as the localization error in the location result evaluation. In Figure 12, the localization errors of the leakage sources are 2.05 cm, 3.03 cm, 3.44 cm, and 1.64 cm, which prove that the method has good robustness. By this method, leakage location experiments are carried out at various points in Figure 6, and the orientation results of the subarray and the localization error of the U-array are shown in Table 4.

It can be seen from the table that the average errors of subarray location results are 3.47° and 4.47°, and the average error of localization of leakage sources by the proposed method is 2.21 cm. The results show that the proposed method can effectively solve the problems caused by the anisotropy and frequency dispersion phenomena of composite structures and effectively locate the gas leakage signal.

It can be seen from Table 4 that the Type2 array has a larger positioning error, which is related to the change in the wave-direction curve of the array. Compared with Type1 and Type3 array curves, the curve of Type2 has a phase shift of 90°. Type1 and Type3 arrays are similar overall, and the positioning errors are relatively concentrated. It can be seen from the orientation error of the subarray that the orientation error of array 2 is greater than that of array 1. From the results of orientation error, when the direction of the leakage source is greater than 90°, its positioning stability will be lower than that below 90°. In addition, when the leakage source direction is located at the boundary of the array measurement range, the orientation error will increase.

**Figure 13:**
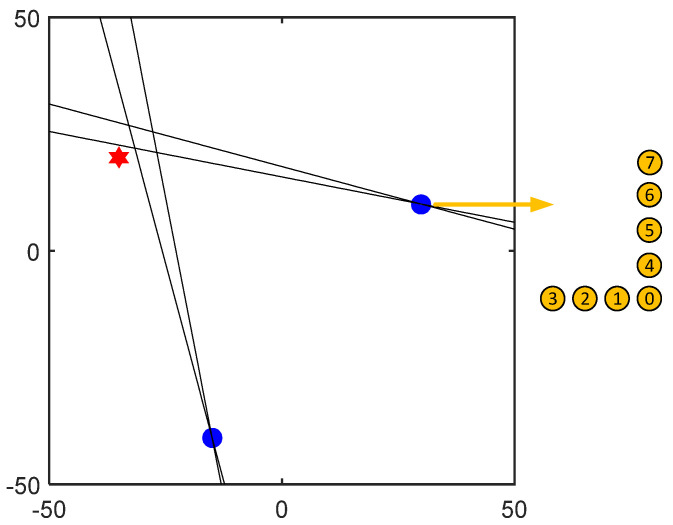
L–array positioning results: The positioning results of Experiment 3 show that the orientation results are 13° and 75°, and the positioning error is 4.53 cm. The leak source is marked with a red five-pointed star, and the reference position of the array is marked with a blue circle.

To verify the advantages of the U-array method, an L-array is used to locate the leakage source in the experiment. Since an L-array can only scan and orient the acoustic source in the range of 0–90°, it is necessary to use two L–arrays to locate the leakage source. Three groups of positioning experiments were carried out in the experiment. Taking the positioning result of Experiment 3 as an example, the positioning result is shown in Figure 13.

The array position and leakage source location results of the comparative experiment are shown in Table 5.

According to the positioning results of the L-array experiment, the positioning error of the L-array positioning method is 3.91 cm, which is larger than the U-array positioning method. In addition, this method needs to arrange two groups of L-arrays and 16 sensors in hardware, which is complicated in the arrangement of structure, while the U-array only needs 12 sensors, and the distribution positions are concentrated. Additionally, the scanning range of a single L-array is 0–90°, while the U-array can scan in the direction of 0–180°. Therefore, the detection effect of the U-array is better than that of the L-array method.

## 5. Discussion and Outlook

This paper proposes a beamforming algorithm based on a U-shaped array to solve the localization problem of continuous leakage acoustic sources in composite plate-like structures. This method realizes the orientation of the leakage source through two sub-arrays of the U-shaped array, solves the Lamb wave dispersion problem in the plate structure through the method of optimal narrow band filtering, and constructs the wave velocity-direction function curve corresponding to the narrow frequency band according to the frequency dispersion curve in different directions to solve the problem of anisotropy of the composite material, which has good performance in the application. The above scheme was verified experimentally, and leakage from a 3 mm aperture in the composite plate was tested. The results show that the proposed U-shaped array beamforming algorithm can obtain a location error of 2.21 cm. The above results can be used for leakage source detection of spacecraft-related structures, which provides a new idea for the structural health detection of spacecraft.

The above experiments verify the effectiveness of the beamforming algorithm for the U-shaped array proposed in this paper, but there are still some problems with the method that need to be improved in the future.

In this paper, a circular leak hole with a 3 mm aperture was used. In the subsequent study, different types of leakage holes will be added for leakage experiments to verify the method.

It can be seen from the location results that in the orientation process of the subarray, when the direction of the leakage source is obtuse, the orientation effect of the array is worse than when the direction is acute, and the orientation treatment in the 90–180° direction will be optimized later.

The positioning error of the Type2 array is larger than that of the other two types, which is related to the lay-up mode of the composite plate and the wave-direction curve. This direction will be optimized in the future to improve positioning accuracy.

Compared with the two L-shaped arrays, the U-shaped array can match a smaller number of sensors, thus reducing the number of sensors. The positioning error of the U-array is better than that of the double-L-array, and a better orientation effect is achieved.

In practice, composite structures will be more complex. The effectiveness of the compensation method of U-array beamforming also deserves attention and study.

In addition, when the aperture of the leakage hole changes, the amplitude and spectrum of the signal will change, but in the process of signal processing, it is necessary to select the filter frequency band through the frequency domain characteristics, so the change of the aperture will not have a great impact on the positioning algorithm.

In conclusion, for continuous leakage, the U-array beamforming algorithm achieves good leak source localization in the composite structure. In subsequent development, optimization and further testing are required for real-world applications.

## Figures and Tables

**Table 1:** The performance parameters of the composite plate.

Parameter	Parameter Values	Units
Density	1560	Kg/m^3^
Young’s modulus	{132 × 10^9^, 8.8 × 10^9^, 8.8 × 10^9^}	Pa
Poisson’s ratio	{0.3115, 0.33, 0.315}	1
Shear modulus	{4.95 × 10^9^, 3.35 × 10^9^, 4.95 × 10^9^}	N/m^2^

**Table 2:** Sub-array position and orientation results.

Experiment Number	Distance	Array 1 #0	Direction/°	Array 2 #0	Direction/°
1	58.3	(15, −10)	31	(−10, −10)	126
2	70.7	(15, −30)	45	(−10, −30)	116
3	40.3	(0, 0)	30	(−20, 0)	124
4	58.3	(−5, −30)	59	(−30, −30)	95
5	56.6	(5, −20)	45	(−20, −20)	110
6	63.2	(−15, −40)	71	(−40, −40)	85
7	63.2	(25,0)	18	(0, 0)	150
8	56.6	(−25, −40)	80.5	(−50, −40)	76.2
9	100	(45, −40)	36.8	(20, −40)	137.5
10	63	(25, 0)	18.4	(0, 0)	150.2
11	60.4	(20, −5)	112.1	(20, −25)	50.7
12	60.2	(5, −25)	138.1	(5, −50)	29.8
13	40.3	(5, 25)	7.2	(−20, 25)	171.6
14	47.2	(5, 45)	32.1	(−20, 45)	59.1
15	74.3	(35, 45)	119.1	(10, 45)	19.6

**Table 3:** Wave velocity distribution at 0° and 90°.

Filter Frequency Band (Hz)	X-axis Wave Speed (m/s)	Y-axis Wave Speed (m/s)
40–60 k	1346	927
60–80 k	1484	1036
80–100 k	1578	1113
100–120 k	1644	1172

**Table 4:** Statistics of subarray orientation results.

Experiment Number	Actual Direction/°	Location Result/°	Actual Direction/°	Location Result/°	Location Error/cm
1 (type 1)	31	32	126	123	3.03
2 (type 1)	45	44	116	111	2.05
3 (type 1)	30	32	124	120	3.52
4 (type 1)	59	59	95	98	1.02
5 (type 1)	45	42	110	108	0
6 (type 1)	71	69	85	86	2.80
7 (type 1)	18	20	150	146	0.92
8 (type 1)	81	77	76	75	1.18
9 (type 1)	37	36	138	132	1.80
10 (type 1)	18	20	150	146	2.74
11 (type 2)	112	114	51	48	3.44
12 (type 2)	138	135	30	28	3.42
13 (type 3)	7	11	172	166	3.64
14 (type 3)	32	34	59	57	1.64
15 (type 3)	19	23	29	31	1.90
Average error		3.47°		4.47°	2.21 cm

**Table 5:** Positioning results of the L-array method.

Experiment Number	Direction 1/°	Location Result 1/°	Direction 2/°	Location Result 2/°	Location Error/cm
1	60	55	20	23	6.64
2	31	29	45	44	0.54
3	10	13	72	75	4.53
Average error					3.91

## Data Availability

The data presented in this study are not publicly available at this time but may be obtained upon reasonable request from the authors.
